# Prevalence and antimicrobial drug resistance of gram-negative bacteria in dairy feed and water: a One Health concern

**DOI:** 10.3389/fvets.2025.1654200

**Published:** 2025-09-24

**Authors:** Safia Arbab, Hanif Ullah, Weiwei Wang, Abdul Qadeer, Abdullah A. Aseeri, Fuad M. Alzahrani, Khalid J. Alzahrani, Khalaf F. Alsharif, Jiyu Zhang

**Affiliations:** ^1^Key Laboratory of Veterinary Pharmaceutical Development, Ministry of Agriculture, Lanzhou, China; ^2^Key Laboratory of New Animal Drug Project of Gansu Province, Lanzhou, China; ^3^Lanzhou Institute of Husbandry and Pharmaceutical Sciences, Chinese Academy of Agricultural Sciences, Lanzhou, China; ^4^Medicine and Engineering Interdisciplinary Research Laboratory of Nursing & Materials/Nursing Key Laboratory of Sichuan Province, West China Hospital, West China School of Nursing, Sichuan University, Chengdu, Sichuan, China; ^5^Department of Cell Biology, School of Life Sciences, Central South University, Changsha, China; ^6^Department of Clinical Laboratory Sciences, College of Applied Medical Sciences, King Khalid University, Abha, Saudi Arabia; ^7^Department of Clinical Laboratories Sciences, College of Applied Medical Sciences, Taif University, Taif, Saudi Arabia

**Keywords:** antimicrobial resistance, antibiotic resistance index, One Health, farm-level surveillance, hygiene practices, regulatory measures

## Abstract

**Introduction:**

Dairy animals are continually at risk of infection due to exposure to contaminated environments, particularly through feed and water. The presence of *Salmonella* spp. and *Escherichia coli* in these sources poses a serious One Health concern due to their potential for antimicrobial resistance (AMR) and subsequent transmission to humans, animals, and the environment. This study was conducted to evaluate the prevalence of these pathogens and their resistance patterns in dairy farm feed and water samples.

**Materials and methods:**

A total of 160 samples, comprising 98 feed and 49 water samples, were collected from dairy farms. Isolates were identified using Gram staining, motility testing, and endospore staining, followed by confirmation with standard biochemical tests (IMViC). Antimicrobial susceptibility testing was carried out, and Multiple Antibiotic Resistance Index (MARI) values were calculated.

**Results:**

Of the 144 analyzable samples, 76 (51.7%) tested positive for E. coli and 68 (46.3%) for *Salmonella* spp. *E. coli* showed the highest susceptibility to ampicillin, cefotaxime, and ciprofloxacin (19%), while *Salmonella* spp. demonstrated the highest susceptibility to cefpodoxime and ampicillin (17%). MARI values exceeding the 0.2 threshold were observed in 6 (7.8%) *E. coli* isolates and 4 (5.8%) *Salmonella* spp. isolates, suggesting high antibiotic exposure. The mean inhibition zones were 9.87 ± 6.16 mm for *E. coli* and 8.5 ± 5.34 mm for *Salmonella* spp., with minimal variation between the two species.

**Conclusion:**

The comparable prevalence and resistance patterns of *E. coli* and *Salmonella* spp. in dairy farm feed and water highlight the risk of antimicrobial-resistant bacteria dissemination across human, animal, and environmental domains. These findings underscore the importance of integrated monitoring systems, judicious antibiotic use, and coordinated stewardship measures within the One Health framework.

## 1 Introduction

The widespread emergence of antimicrobial resistance (AMR) is a growing global health threat that extends beyond human medicine to encompass animal and environmental health. Ensuring the safety of animal feed and drinking water is critical, as contaminated sources can act as reservoirs and transmission routes for resistant pathogens, with major consequences for animal production, public health, and the economy (1). However, several welfare concerns remain unaddressed. Early weaning, still a widespread practice in the dairy industry, has been a longstanding challenge in recent years (2).

Animal feed and byproducts are frequently contaminated with bacterial pathogens, including *Salmonella enterica, Staphylococcus aureus, Listeria monocytogenes, Clostridium spp., Aeromonas*, and *Campylobacter*, with *Salmonella spp*. being the most detected (3). Such contamination poses additional risks in regions where surveillance systems are limited and antimicrobial use in farming is poorly regulated, such as in low- and middle-income countries like Pakistan (4–6).

Antimicrobials are essential for treating infections in both humans and animals; however, their excessive or inappropriate use disrupts natural microbiota and drives the selection of multidrug-resistant (MDR) bacteria (7, 8). While these agents reduced infectious disease mortality in the 20th century, their widespread use, particularly in livestock production, often at sub-therapeutic doses, has contributed to the global rise of resistance (9–12). Recent reports highlight the detection of resistant *Escherichia coli* and *Salmonella spp*. in food-producing animals and animal feed, underscoring the role of feed and water as potential sources and reservoirs of resistance (13). Regardless of its origin, antibiotic resistance has been increasing, and it is now projected to become one of the leading causes of death, potentially contributing to more than 10 million fatalities each year (14).

Recent incidents involving these bacterial isolates in companion animals and livestock have highlighted the need for increased monitoring studies in livestock (15). *E. coli* strains that produce β-lactamase enzymes are increasingly detected in food-producing animals, posing a potential risk as sources of infection or reservoirs that contribute to the transmission of these harmful bacteria (16). *Salmonella* spp. and *E. coli* have been found in animal feed in recent studies, and their AMR profiles have also been characterized (17).

The One Health approach provides a valuable framework to address this challenge, recognizing the interconnectedness of human, animal, and environmental health (18). Monitoring resistant Gram-negative bacteria in dairy feed and water is therefore critical to identify contamination pathways, inform stewardship strategies, and mitigate the spread of AMR in agricultural systems.

Accordingly, this study aimed to investigate the prevalence and antimicrobial resistance profiles of Gram-negative bacteria in dairy animal feed and drinking water. These microorganisms pose risks to both veterinary care and public health, as they can infect animals, contaminate feed and water, and serve as carriers of resistance genes, particularly in settings where hygiene and antimicrobial oversight remain limited.

## 2 Materials and methods

### 2.1 Ethics statement

Animal research was guided by the Guide for the Care and Use of Laboratory Animals, released by the Ministry of Science and Technology of the People's Republic of China, with all efforts made to decrease animal suffering. This study was funded by grants from the National Natural Science Foundation of China and the Agricultural Research System (CARS-37).

### 2.2 Sample collection

#### 2.2.1 Feed samples

A total of 98 feed samples were collected from animal farms under aseptic conditions with assistance from farm staff and agricultural distributors. Sub-samples were proportionally selected according to the number of units in each batch, following established guidelines. Each sample was labeled, placed in sterile bags, and transported to the laboratory for analysis.

#### 2.2.2 Drinking water samples

Forty-nine drinking water samples were aseptically collected from dairy animal troughs using sterile disposable containers. Samples were immediately placed in insulated boxes with ice packs and transported to the laboratory within 4 h for microbiological analysis.

#### 2.2.3 Sample size justification

A total of 160 samples (98 feed and 49 water) were collected to ensure adequate representation across the sampled farms while remaining feasible given available resources. The sample size is consistent with previous studies investigating bacterial contamination in livestock feed and water.

### 2.3 Screening and isolation of microorganisms

Isolation of *Salmonella* spp. and *Escherichia coli* was performed following standard microbiological protocols (19). Primary inoculation was carried out on nutrient agar, MacConkey agar, eosin methylene blue (EMB) agar, *Salmonella-Shigella* (SS) agar, mannitol salt agar, and blood agar. Plates were incubated aerobically at 37 °C for 24 h. Colony morphology, Gram staining, and motility (hanging drop method) were assessed for preliminary identification.

#### 2.3.1 Primary isolation and culture

Swab samples were inoculated onto nutrient agar, MacConkey agar, eosin methylene blue (EMB) agar, *Salmonella/Shigella* (SS) agar, mannitol salt agar (MSA), and blood agar, and incubated aerobically and anaerobically at 37 °C for 24 h. Colony characteristics were recorded, and Gram-stained smears were prepared for preliminary identification (20).

#### 2.3.2 Sub-culturing and identification

Suspicious colonies were further purified by repeated sub-culturing onto selective and differential media. Pure isolates were confirmed by colony morphology, Gram reaction, and biochemical tests (catalase, oxidase, indole, coagulase, TSI, Simmons' citrate), as well as the analytical profile index (API).

For *E. coli*, lactose-fermenting colonies showing a green metallic sheen on EMB or pink colonies on MacConkey agar were further confirmed using API and standard biochemical assays. For *Salmonella* spp., colonies appearing colorless and transparent on SS agar were confirmed by API, biochemical assays, and Gram staining (8).

### 2.4 Microscopic analysis

#### 2.4.1 Motility test (hanging drop method)

A loopful of inoculum from an isolated colony of each sample was prepared and examined under the microscope using the hanging drop technique.

#### 2.4.2 Gram's staining

Gram staining was performed following the standard protocol recommended by the American Society for Microbiology (ASM) (21).

### 2.5 Biochemical tests

#### 2.5.1 IMViC test

##### 2.5.1.1 Indole test

Pure bacterial culture was introduced into tryptophan broth (HIMEDIA^®^ Ref: M1339-500G) and incubated at 37 °C for 24 h. After incubation, Kovac's reagent (2–3 drops) was added to each tube, and the reaction was examined after 15 min (22).

##### 2.5.1.2 Methyl-Red test

MR-VP broth (HIMEDIA^®^ Ref: M070-500G) was inoculated with the test organism and incubated at 37 °C for 24 h. After incubation, 2–3 drops of methyl red indicator were added, and observations were made after 15 min (22).

##### 2.5.1.3 VP test

Five test tubes, each labeled with a specific sample ID, were filled with VP broth (HIMEDIA^®^ Ref: M070F-500G), inoculated with the respective cultures, and incubated at 37 °C for 24 h. After incubation, 2–3 drops of Barritt's reagent were added to each tube, and results were noted after 15 min.

##### 2.5.1.4 Citrate test

The citrate utilization test was conducted using citrate agar slants (HIMEDIA^®^ Ref: M099-500G). Bacterial cultures were streaked along the surface of the slants, and the tubes were incubated at 37 °C for 24 h (21).

### 2.6 Antibiotic susceptibility test

Antimicrobial susceptibility testing of the isolates was carried out using Mueller-Hinton agar following the modified Kirby-Bauer disc diffusion technique, based on Clinical Laboratory Standards Institute (CLSI) recommendations. Each bacterial isolate was evenly spread on separate nutrient agar plates, and antibiotic discs were placed onto the surface using a sterile applicator to ensure proper contact. The plates were incubated at 37 °C for 24 h. To detect extended-spectrum β-lactamase (ESBL) production, the double-disc synergy test was performed using cefotaxime and amoxicillin-clavulanate discs (23).

A total of ten antimicrobial agents were evaluated: ampicillin (AMC) 10 μg, augmentin (AUG) 30 μg, cefotaxime (CTX) 30 μg, cefpodoxime (CP) 10 μg, ceftazidime (CAZ) 30 μg, amoxicillin (AMX) 25 μg, cefuroxime (CXM) 30 μg, ciprofloxacin (CPX) 10 μg, tetracycline (TE) 30 μg, and streptomycin (STR) 10 μg. Interpretation of the inhibition zones was carried out following the Clinical Laboratory Standards Institute (CLSI) guidelines (23, 24).

### 2.7 Multiple Antibiotic Resistance Index (MARI) determination

The Multiple Antibiotic Resistance Index (MARI) for each isolate was calculated using the formula: MARI $=\;\frac{a}{b}$

Where *a* is the number of antibiotics to which the isolate was resistant, and *b* is the total number of antibiotics tested. An MARI value >0.2 indicates that the isolate likely originated from an environment with high antibiotic use, suggesting potential multiple resistance traits. The 0.2 threshold is widely used in studies and aligns with guidelines from public health agencies to identify high-risk resistance profiles (25).

### 2.8 Statistical analysis

Graphs were generated using Microsoft Office Excel 2007. Descriptive statistical analysis was performed, including calculations of mean, standard deviation, frequencies, and percentages. The data were first assessed for normal distribution to determine the appropriate statistical tests for significance analysis. Mean values were also used to calculate the Multiple Antibiotic Resistance Index (MARI) scores.

## 3 Results

### 3.1 Confirmation of bacterial isolates

A total of 160 samples of water and animal feed were analyzed. Although the number of samples was somewhat limited, the sample size remains substantial. Feed samples (*n* = 98) and animal drinking water samples (*n* = 49) were collected across all targeted animal species (Table 1).

**Table 1:** Prevalence of *Salmonella* spp. and *E. coli* isolates in dairy feed and water samples.

**Species**	**Number of samples**	***E. coli* isolates (Feed)**	***E. coli* Isolates (Water)**	***Salmonella* spp. isolates (Feed)**	***Salmonella* spp. isolates (Water)**
Cattle	45	19 (30.4%)	11 (17.6%)	16 (25.6%)	9 (14.4%)
Buffalo	45	18 (28.8%)	6 (9.6%)	16 (25.6%)	9 (14.4%)
Sheep	35	12 (19.2%)	5 (8%)	9 (14.4%)	3 (4.8%)
Goat	35	5 (8%)	3 (4.8%)	3 (4.8%)	3 (4.8%)

**Figure 1:**
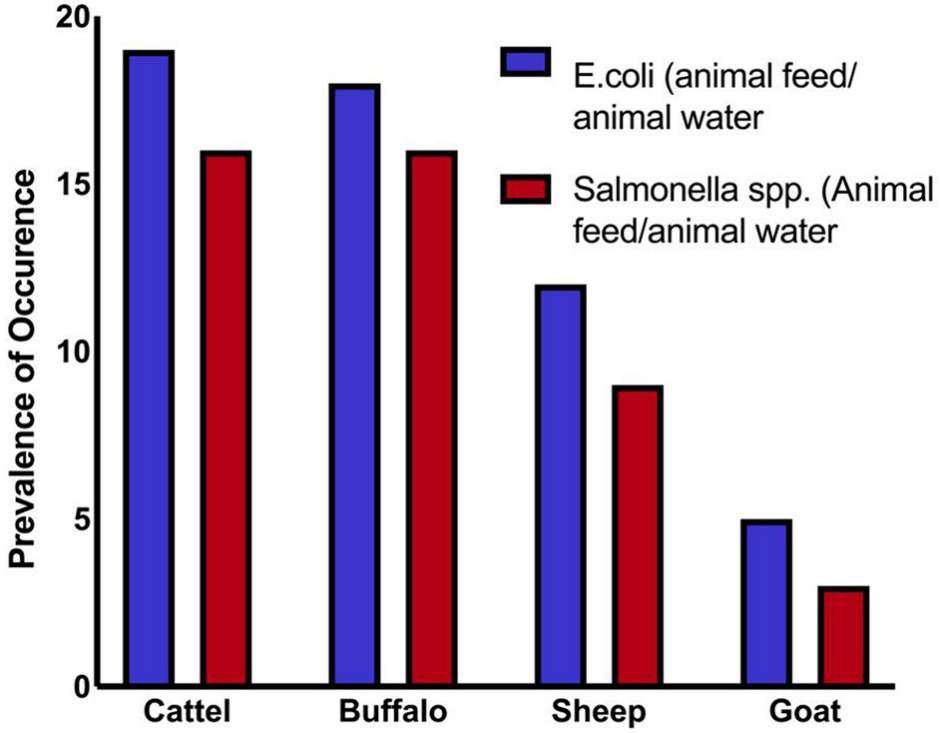
Prevalence of isolates from different microorganisms.

Overall, 71 samples (44.3%) tested positive for *Salmonella* spp., and 79 samples (49.3%) tested positive for *E. coli*. These findings suggest significant contamination at several sampling sites, with *E. coli* being the most frequently identified organism. At every critical sampling point, the levels of pathogenic bacterial contamination in both drinking water and animal feed were notably high. The highest contamination levels were observed in cattle and buffalo feed and water samples, whereas the lowest were found in sheep and goat samples. As shown in Table 1 and Figure 1, the variation in *Salmonella* spp. Isolation rates between farms (critical sampling points) were statistically significant.

### 3.2 Streak plate method

**Figure 2:**
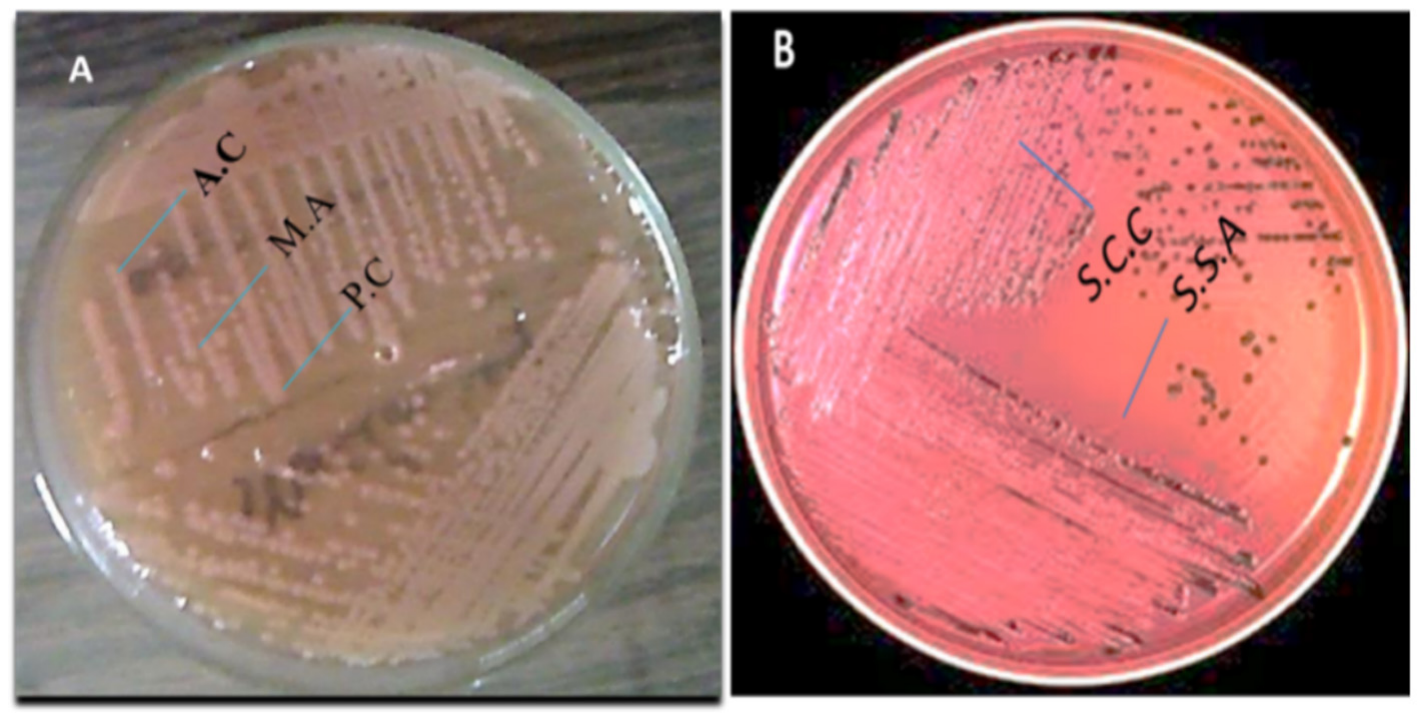
**(A)** *Escherichia coli* colonies and **(B)** *Salmonella* spp. colonies cultured on selective agar plates.

Colony morphology observations indicated the presence of a single bacterial type in the samples. Pigment formation was observed in some plates, as illustrated in Figure 2.

#### 3.2.1 Microscopic analysis

##### 3.2.1.1 Motility test

The motility test results showed that selected bacterial isolates from each sample exhibited motility, and all motile organisms were identified as rod-shaped. Under 10X and 100X magnification in an endospore stain, *E. coli* and *Salmonella* appeared as pink, rod-shaped cells, with no green endospores visible. Since *E. coli* is a non-endospore-forming bacterium, only the vegetative cells will be observed. At 10X, we can see the overall distribution of the bacterial cells, while at 100X, individual pink rods were visible under oil immersion, but no green spore structures were present. Samples appeared pink in color, indicating a Gram-negative nature.

### 3.3 IMViC test results

The IMViC test is a series of biochemical tests used to help identify and differentiate members of the Enterobacteriaceae family, particularly *Escherichia coli* (*E. coli*), from other coliforms and enteric bacteria, such as *Salmonella*. The results of the IMViC biochemical tests are summarized in Table 2.

**Table 2:** IMViC biochemical tests for identifying *E. coli* and *Salmonella* spp.

**Test**	***E. coli***	***Salmonella* spp**
Indole	Positive (Red)	Negative (No color change)
Methyl Red	Positive (Red)	Positive (Red)
Voges-Proskauer	Negative (No color change)	Negative (No color change)
Citrate	Negative (Green)	Positive (Blue)

#### 3.3.1 Indole test

The indole test is used to assess an organism's ability to break down the amino acid tryptophan and produce indole. Escherichia coli served as the positive control, while Bacillus subtilis was used as the negative control. In the positive control, a cherry-red ring developed at the surface of the medium, whereas no color change was observed in the negative control. A pink to red layer appeared in the test samples above the medium, indicating a positive indole reaction. These results suggest that all the bacterial isolates tested could produce indole.

#### 3.3.2 Methyl Red test (MR)

The Methyl Red (MR) test assesses an organism's ability to perform mixed-acid fermentation of glucose, leading to the formation of stable acidic end products. The procedure involves inoculating the test organism into MR-VP broth, followed by incubation at 37 °C for 24 to 48 h. After incubation, Methyl Red indicator is added. A red coloration signifies a pH below 4.4, indicating a positive result for mixed-acid fermentation. In contrast, a yellow or orange color suggests a higher pH and a negative result.

#### 3.3.3 Voges-Proskauer test

The Voges-Proskauer (VP) test detects the production of acetoin, a neutral intermediate formed during butylene glycol fermentation of glucose. This test identifies whether an organism can ferment glucose to produce acetyl methyl carbinol (acetoin). Escherichia coli was used as both the positive and negative control for comparison. Upon analysis, the appearance of a red or pink color indicated a positive result. Based on the observed results, all tested samples showed a positive VP reaction, suggesting that they produce acetoin during glucose fermentation.

#### 3.3.4 Citrate utilization test

Determines the ability of an organism to use citrate as its sole carbon source, converting it into alkaline products (such as ammonia). Inoculate the bacteria onto Simmons' Citrate Agar (or another citrate medium). Incubate at 37 °C for 24–48 h. A color change from green to blue, indicating alkalinization due to citrate utilization. No color change (the medium remains green), indicating the organism cannot utilize citrate. The results of the IMViC biochemical tests are summarized in Table 2.

The IMViC test series is a simple and reliable method for identifying and differentiating enteric bacteria based on their biochemical characteristics. By analyzing the results, laboratories can classify organisms, facilitating accurate identification, especially in clinical microbiology.

### 3.4 Antimicrobial susceptibility of the bacterial isolates

**Figure 3:**
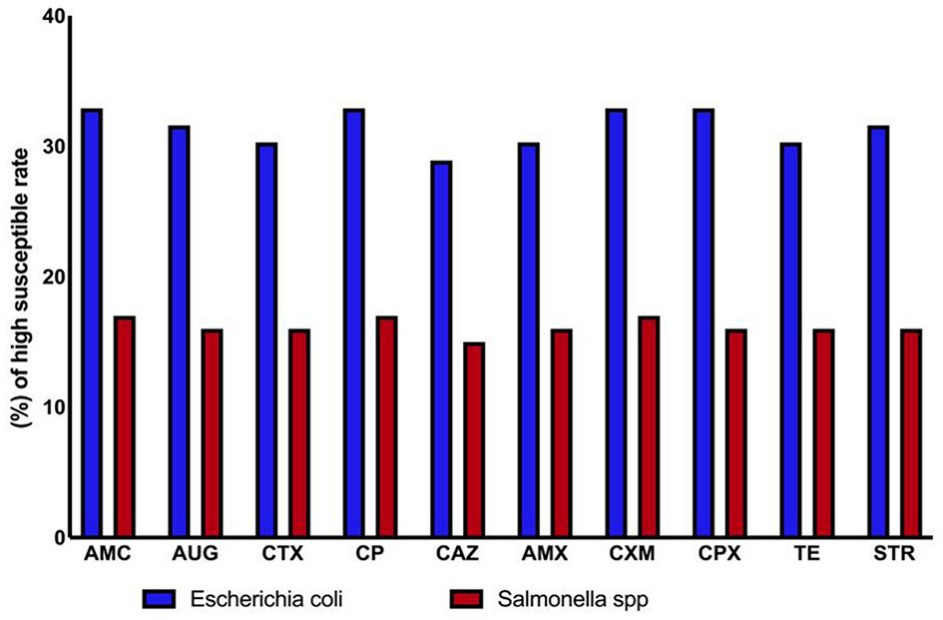
High susceptibility rates in antibiotic-resistant *Escherichia coli* and *Salmonella* spp.

Antibiogram analysis indicated that antibiotics were generally effective against both Gram-positive and Gram-negative bacteria. However, *Salmonella* spp. and environmental *E. coli* strains isolated from drinking water and animal feed showed signs of antibiotic resistance. Resistance to ten different antibiotics was detected among the isolates, suggesting widespread contamination with varying resistance profiles. Overall, *E. coli* was the most frequently isolated bacterium, while *Salmonella* spp. demonstrated relatively high antibiotic susceptibility (Tables 3, 4, and Figure 3).

**Table 3:** Antibiotic sensitivity of gram-negative *E. coli* (*n* = 76) isolates from animal feed and water samples.

**Antimicrobial**	**Susceptible (No, %)**	**Intermediate (No, %)**	**Resistant (No, %)**
AMC	25 (32.9%)	0 (0%)	1 (1.3%)
AUG	24 (31.6%)	0 (0%)	1 (1.3%)
CTX	23 (30.3%)	0 (0%)	2 (2.6%)
CP	25 (32.9%)	2 (2.6%)	0 (0%)
CAZ	22 (28.9%)	0 (0%)	0 (0%)
AMX	23 (30.3%)	0 (0%)	0 (0%)
CXM	25 (32.9%)	7 (9.2%)	2 (2.6%)
CPX	25 (32.9%)	4 (5.3%)	2 (2.6%)
TE	23 (30.3%)	1 (1.3%)	0 (0%)
STR	24 (31.6%)	0 (0%)	1 (1.3%)

**Table 4:** Antibiotic sensitivity of gram-negative bacterial *Salmonella* spp. (*n* = 68) isolates from animal feed and water samples.

**Antimicrobial**	**Susceptible (No, %)**	**Intermediate (No, %)**	**Resistance (No, %)**
AMC	25 (17%)	0 (0%)	1 (1.3%)
AUG	24 (16.3%)	0 (0%)	1 (1.3%)
CTX	23 (15.6%)	0 (0%)	1 (1.3%)
CP	25 (17%)	2 (1.3%)	0 (0%)
CAZ	22 (14.9%)	0 (0%)	0 (0%)
AMX	23 (15.6%)	0 (0%)	0 (0%)
CXM	20 (17%)	7 (4.7%)	0 (0%)
CPX	24 (16.3%)	4 (2.7%)	2 (1.3%)
TE	23 (15.6%)	1 (1.3%)	0 (0%)
STR	24 (16.3%)	0 (0%)	1 (1.3%)

AMC, ampicillin; AUG, Augmentin; CTX, cefotaxime; CP, cefpodoxime; CAZ, ceftazidime; AMX, amoxicillin; CXM, cefuroxime; CPX, ciprofloxacin; TE, tetracycline; STR, streptomycin.

### 3.5 Resistance rates and Multiple Antibiotic Resistance Index (MARI) of bacterial isolates

The susceptibility of bacterial isolates to 10 antibiotics was assessed by measuring inhibition zone diameters. Both *E. coli* and *Salmonella* spp. demonstrated resistance to multiple antibiotics. Specifically, *E. coli* isolates were resistant to six antibiotics, while *Salmonella* spp. Isolates were resistant to four. Both species remained highly susceptible to ampicillin, cefpodoxime, cefotaxime, ciprofloxacin, Augmentin, and streptomycin (Table 5).

**Table 5:** Multidrug resistance indexes (MARIs) of the isolates.

**Isolates**	**List of antibiotics (phenotype)**	**MRI**
*E. coli*	AMC, AUG, CTX, CXM, CPX, STR	6
*Salmonella* spp	AMC, AUG, CTX, CPX	4

Multidrug resistance was evaluated using the Multiple Antibiotic Resistance Index (MARI), calculated as the ratio of the number of antibiotics to which an isolate was resistant to the total number tested (*n* = 10). *E. coli* isolates exhibited a higher MARI (0.6), indicating exposure to environments with high antibiotic use and potential multi-resistance, whereas *Salmonella* spp. showed moderate resistance with a MARI of 0.4.

Table 6 shows the mean ± standard deviation (SD) of inhibition zone diameters for each species. *E. coli* demonstrated slightly higher resistance levels compared to *Salmonella* spp., although differences were minimal. These findings highlight the presence of multidrug-resistant bacteria in the sampled feed and water source.

**Table 6:** Bacterial organisms isolated with different means and standard deviations.

**Bacterial isolates**	**Mean ±SD**
*E. coli*	9.87 ± 6.16
*Salmonella* spp.	8.5 ± 5.34

## 4 Discussion

Dairy animals are extensively studied in animal welfare, yet challenges such as early weaning persist (2, 26). In this study, we analyzed the prevalence and multidrug resistance (MDR) patterns of *Salmonella* spp. and *E. coli* in water and animal feed samples. *E. coli* was the most frequently isolated species (51.7%), followed by *Salmonella* spp. (46.3%), consistent with previous reports in livestock and feed (27).

Our findings revealed resistance to multiple beta-lactam antibiotics in both *E. coli* and *Salmonella*, with intermediate resistance observed for ciprofloxacin, tetracycline, cefixime, and cefuroxime. These patterns align with studies from Pakistan and neighboring countries, highlighting the widespread presence of MDR pathogens in livestock environments (28), these results are consistent with those of the current investigation. Similarly, high levels of *E. coli* were found in Jordanian cows (29). These findings are consistent with recent studies that identified *E. coli* as the most common species in healthy animals (30).

A series of biochemical tests was conducted to characterize the bacterial isolates from the samples. The hanging drop technique was employed to observe bacterial motility, as well as to examine the size, shape, and cellular arrangement of the organisms (31).

The IMViC test provides a useful method to differentiate *E. coli* from *Salmonella*. While both are gram-negative, facultative anaerobes, *E. coli* tends to be indole-positive, methyl red-positive, and citrate-negative, while *Salmonella* is indole-negative, methyl red-positive, and citrate-positive. The differences in citrate utilization are particularly key, as *Salmonella* can thrive on citrate, whereas *E. coli* cannot. These tests help in confirming the identity of these pathogens in microbiological investigations (32, 33). Previous research has shown that extensive investigations into the biochemical characteristics of bacteria have been conducted to develop biochemical typing systems, which play a crucial role in the epidemiological tracking and identification of bacterial strains (34).

The VP test determines whether organisms produce acetyl methyl carbinol by fermenting glucose (35). *Bacillus subtilis* and *E. coli* were used as the positive and negative controls, respectively, for the Voges-Proskauer (VP) test. Upon analysis of the test results, no development of the characteristic red or pink color was observed in any of the samples, indicating that all tested isolates were negative for acetoin production.

The antibiotics with the highest sensitivity rates in *E. coli* isolates in this study were ampicillin, cefpodoxime, cefotaxime, and ciprofloxacin (25 and 19%), followed by streptomycin and Augmentin (24 and 18.4%). These findings align with previous studies, which have reported a rapid increase in antibiotic resistance, particularly in *E. coli* strains isolated from animals (36, 37). The highest resistance to doxycycline was observed in *E. coli*. In comparison, resistance rates for gentamicin were 66%, tetracycline 72%, ampicillin 85%, and amoxicillin + clavulanic acid 58% (25, 38).

However, *Salmonella s*pp. had the highest rate of amoxicillin resistance in this study 50%. Several researchers from Ethiopia reported antibiotic-resistant *Salmonella* isolates from feed in previous studies (39), and from other countries. A previous study reported that *Salmonella spp*. isolates exhibited resistance to nalidixic acid 78.57%, tetracycline 42.58%, and ampicillin 42.58% (40); Similarly, a large percentage of Salmonella isolates, 100% were shown to be resistant to ampicillin in another investigation (8, 41).

The highest number of isolates for both *Salmonella spp*. and *E. coli* was found in the animal feed and water. This type of feed is intended for direct consumption, but its production is often less regulated compared to other forms of feed and may not adhere to commercial standards. The feed is typically produced through mechanical homogenization, with high temperatures rarely used. Potential contributing factors to contamination include inadequate cleaning of production lines, poor rodent control, improper storage on floors rather than in designated food storage areas, and substandard packaging and storage conditions (42, 43).

The Multiple Antibiotic Resistance Index (MARI) among isolates ranged from 0.06 to 0.07, indicating moderate exposure to antibiotics, in contrast to earlier overstated values. High MARI values, even at this level, suggest ongoing selective pressure due to antibiotic use in farms and underscore the risk of MDR dissemination through feed and water (44).

Our findings of an average MARI of 0.8 exceed those reported in similar studies in Ghana (0.11– 0.78) and South Africa (0.3–0.6), further underscoring the severity of antibiotic misuse in the sampled farms. These elevated MARI values not only pose a threat to animal health but also carry significant (20). One Health implications, as they indicate a heightened risk of antimicrobial resistance transmission among animals, humans, and the environment (45).

Contamination in feed and water likely arises from inadequate hygiene during production, storage, and handling, consistent with previous reports. These findings emphasize the importance of strict biosecurity measures, proper feed management, and monitoring of antibiotic use to prevent the spread of resistant bacteria (46).

Comparisons with global surveillance programs, including WHO and national monitoring systems, show similar resistance trends in *E. coli* and *Salmonella* isolates from food-producing animals (47, 48). This underscores the One Health implications of antimicrobial resistance, highlighting potential transmission between animals, humans, and the environment. It has documented high levels of multidrug resistance in *E. coli* and *Salmonella* from food-producing animals in several regions. Similarly, the U.S. National Antimicrobial Resistance Monitoring System (30, 49) reports comparable resistance trends, and national surveillance reports from Pakistan (2022) and China (2023) show elevated MARI values and widespread resistance to beta-lactam and tetracycline-class antibiotics in livestock environments (50, 51).

Although our sample size was sufficient, the relatively small number of positive samples limited additional statistical analyses. Future studies should include molecular characterization of resistance genes to better understand the mechanisms underlying MDR in livestock environments.

In conclusion, our results highlight the prevalence of *E. coli* and *Salmonella* in dairy feed and water, their multidrug resistance patterns, and the need for improved antimicrobial stewardship and hygiene practices in livestock production.

## 5 One Health integration

Our study highlights the interconnectedness of animal, environmental, and human health through the lens of antimicrobial resistance (AMR). The presence of resistant *E. coli* and *Salmonella* in animal feed and water not only affects livestock health but also poses risks to humans, particularly farm workers who have direct occupational exposure to these bacteria. Moreover, consumption of contaminated milk and dairy products can be a direct route for transmitting resistant pathogens to consumers, posing a significant public health concern.

To address these challenges, we propose a One Health surveillance model that includes regular monitoring of livestock health and bacterial resistance patterns, environmental sampling of soil and water runoff to detect contamination sources, and, where ethically permissible, screening of human handlers for colonization or infection with resistant bacteria. This integrated approach can enhance early detection, improve risk assessment, and inform coordinated interventions to control the spread of AMR in the region.

## 6 Significance and future directions

This study highlights the high prevalence of multidrug-resistant *E. coli* and *Salmonella* in dairy animal feed and water, underscoring the urgent need for targeted interventions to reduce antimicrobial misuse in livestock production. Future work should include molecular characterization of these isolates, such as detection and sequencing of specific resistance genes, to better understand the genetic determinants and potential for horizontal gene transfer. Longitudinal studies are essential to monitor resistance trends over time and assess the effectiveness of intervention strategies. Expanding surveillance to include environmental sources such as soil, manure, and farm runoff will provide a more comprehensive picture of AMR dissemination within and beyond farm boundaries. These findings also have important policy implications, supporting the development of farm-level AMR monitoring programs and strengthened antimicrobial stewardship frameworks that align with national and global One Health initiatives.

## 7 Conclusion

The high prevalence of antimicrobial-resistant Gram-negative bacteria in dairy animal feed and water poses a serious threat to both animal and public health. This resistance is likely driven by factors such as poor sanitation, overuse of antibiotics, and environmental contamination. The detection of multidrug-resistant strains highlights the urgent need for stricter regulations on antibiotic use in livestock farming. To address this challenge, regular monitoring, improved hygiene, antimicrobial stewardship programs, and sustainable alternatives to antibiotics should be implemented. Strengthening policies and veterinary diagnostic capabilities, along with raising public awareness about responsible antibiotic use, are essential steps to reduce antimicrobial resistance. Future research should focus on understanding resistance mechanisms and developing effective control strategies. Immediate action is crucial to safeguard animal welfare and prevent the spread of antimicrobial resistance.

## Data Availability

The original contributions presented in the study are included in the article/supplementary material, further inquiries can be directed to the corresponding author.
